# Right ventricular function assessment by cardiac MRI as predictor of outcomes in Coronary Artery Bypass Graft surgery

**DOI:** 10.1186/1532-429X-13-S1-P184

**Published:** 2011-02-02

**Authors:** Krishna Kancharla, Abdalla Elagha, Peter C Hill, Steven W Boyce, Anthon R Fuisz, Gaby Weissman

**Affiliations:** 1 Washington Hospital Center, Washington, DC, USA

## Objective

The aim of this study is to identify if right ventricular function (RVF) can predict short term outcomes after coronary artery bypass graft (CABG).

## Background

RVF is not part of traditional CABG risk prediction models. Cardiac magnetic resonance (CMR) is the gold standard test for RVF. We studied the relation between RVF and CABG outcomes.

## Methods

All patients from 01/2003 to 2/2010 who had CMR ≤30 days prior to CABG were included. RVF was graded as normal (nRVF) or reduced (rRVF). Outcome variables included intensive care unit length of stay ICU-LOS, hospital LOS (H-LOS), 30 day, and long term mortality. Short ICU-LOS was defined as ≤ 24 hours and prolonged ICU-LOS as >48 hours. Short H-LOS was defined as ≤ 5 days and prolonged H-LOS as > 14 days. Society of thoracic surgery (STS) mortality risk scores was calculated. Outcomes were evaluated between the nRVF and rRVF groups using Fisher’s exact test, Mann-Whitney test and logistic regression for statistical analysis.

## Results

202 patients met inclusion criteria. Mean follow up was 3.6 years. Five patients died in the ICU and 9 within 30 days. There was no difference between RVF groups in ICU and <30 day mortality (p=0.67 and 0.72 respectively). Long term mortality was 22% and 28% in the nRVF and rRVF groups respectively (p= 0.38)

Median ICU-LOS was 23 hours and 40.5 hours in the nRVF and rRVF groups respectively (p=0.008), with 57% of nRVF having short ICU-LOS vs. 36% in the rRVF (OR 2.4 (1.3-4.4)). Prolonged ICU-LOS was 22% vs. 37% (p=0.02) in the nRVF and rRVF groups respectively. After adjusting for log transformed STS score, RVF was independently associated with short and prolonged ICU-LOS (p=0.01 and 0.04 respectively)

**Figure 1:**
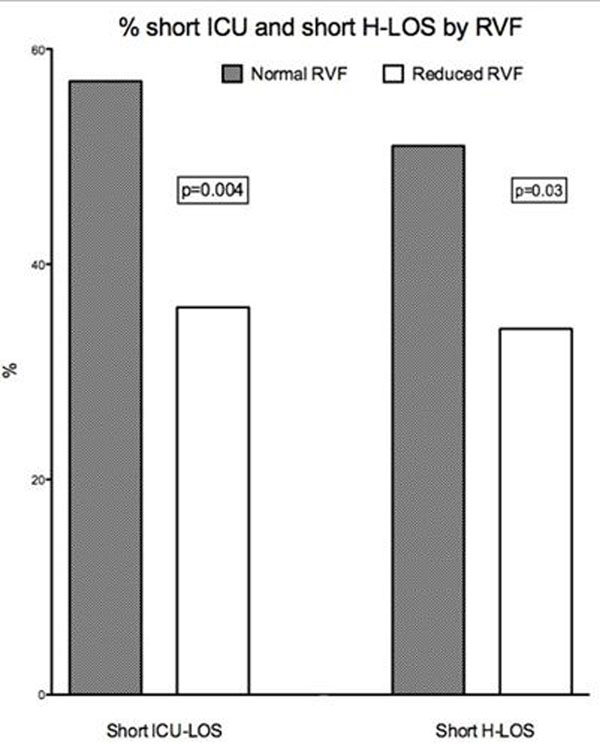


Median H-LOS was 5 days vs. 7 days in the nRVF and rRVF groups respectively (p=0.02) with 51% of nRVF having short H-LOS compared to 34% in the rRVF group (p=0.03). After adjusting for log transformed STS, RVF has a trend towards an independent association with short H-LOS (p=0.09). Figure 1.

## Conclusion

RV function by Cardiac MRI prior to CABG predicts ICU-LOS and H-LOS. RVF does not predict either short term or long term mortality. Adding RVF to current CABG outcome prediction models might improve predictive power.

